# Geometric parameterisation of pelvic bone and cartilage in contact analysis of the natural hip: An initial study

**DOI:** 10.1177/0954411915592656

**Published:** 2015-08

**Authors:** Xijin Hua, Junyan Li, Ruth K Wilcox, John Fisher, Alison C Jones

**Affiliations:** 1 Institute of Medical and Biological Engineering, School of Mechanical Engineering, University of Leeds, Leeds, UK; 2 School of Science and Technology, Middlesex University, London, UK

**Keywords:** Hip, finite element, parameterisation, parameterised models, contact analysis

## Abstract

Parameterised finite element models of the human hip have the potential to allow controlled analysis of the effect of individual geometric features on the contact mechanics of the joint. However, the challenge lies in defining a set of parameters which sufficiently capture the joint geometry in order to distinguish between individuals. In this study, a simple set of parameters to describe the geometries of acetabulum and cartilage in the hip were extracted from two segmentation-based models, which were then used to generate the parameterised finite element models for the two subjects. The contact pressure and contact area at the articular surface predicted from the parameterised finite element models were compared with the results from the segmentation-based models. The differences in the predicted results between the parameterised models and segmentation-based models were found to be within 11% across seven activities simulated. In addition, the parameterised models were able to replicate features of the contact pressure/area fluctuations over the loading cycle that differed between the two subjects. These results provide confidence that the parameterised approach could be used to generate representative finite element models of the human hip for contact analysis. Such a method has the potential to be used to systematically evaluate geometric features that can be captured from simple clinical measurements and provide a cost- and time-effective approach for stratification of the acetabular geometries in the patient population.

## Introduction

There are a growing number of surgical interventions for osteoarthritis of the hip that affect natural cartilage contact mechanics, such as hemi-arthroplasty^1–3^ and treatments for femoroacetabular impingement^4^ where the individual patient anatomy is important. In order to assess these interventions and provide guidelines on patient stratification, it is necessary to be able to characterise geometric features of the joint. Finite element (FE) modelling is an effective tool for analysing the effect of geometric features of the hip on its contact mechanics.^4–7^ FE models can now be generated in a highly subject-specific manner by segmenting geometries from three-dimensional scans.^8,9^ This approach can provide detailed contact mechanics data and facilitate direct validation with experimental measurements in individual patients or specimens.^1,8,10^ However, due to the rigid nature of the image-based subject-specific modelling process, it is difficult to systematically alter geometric features to understand their effect on the contact mechanics.^4,9^ In addition, detailed subject-specific models usually require three-dimensional computed tomography (CT) imaging which is time-consuming and is not standard in the current clinical assessment of osteoarthritis.^11–13^

This work focused on the development of a parameterised model of the natural human hip, where each geometric feature is generated using a series of predefined mathematical or algorithmic steps. A model generation system such as this could be used to rapidly create subject-specific geometries based on a set of measurements taken from a laboratory specimen or from CT images of a patient. The parameterisation of each geometric feature would make it possible to test the sensitivity of hip contact mechanics to an individual aspect, without mitigating factors. The challenge lies in the definition of a set of parameters capable of capturing the joint geometry and distinguishing between individuals in terms of features which affect joint contact mechanics.

The aim of this initial study was to develop and evaluate a new parameterised model of the bone and cartilage on the acetabular side of the hip joint, using a minimal set of parameters and focusing on features affecting the cartilage contact mechanics. A simple set of parameters was generated to describe the shape of the human acetabulum and cartilage, with the broad principle of including more detail closer to the articular surface. These parameters were adjusted to replicate the geometry of two individual hips using measurements taken from three-dimensional CT images. The same images were also used to generate two segmentation-based models, using established image segmentation methods.^1,10^ In order to assess the degree to which the parametric models could replicate the results of the segmentation-based models, FE analysis of the contact mechanics was performed on both the parameterised and segmentation-based models, under seven different daily activities. An additional segmentation-based case was also used to analyse the effect of truncating the pelvic bone on the cartilage contact mechanics of the hip.

## Methods

### Segmentation-based pelvic and acetabular geometries

**Figure 1.**
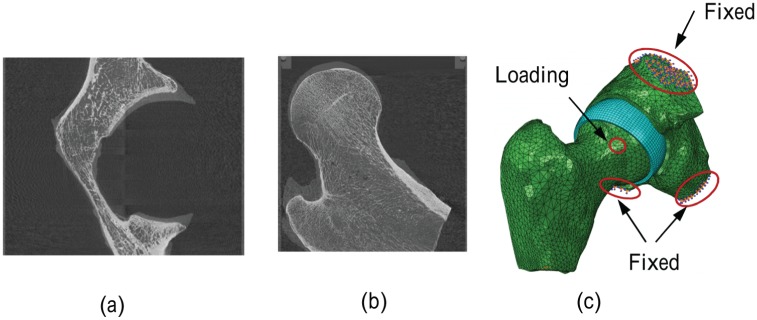
Cadaveric specimen micro CT of (a) pelvic bone, (b) femur bone and (c) model S2 from cadaveric specimen.

Two segmentation-based models of human hip joints were generated from separate image sources. Images of the left pelvic bone and femur of a 38-year-old male, who was healthy at the time of death, were obtained from the BEL repository (website: www.biomedtown.org/biomed_town/LHDL/Reception/datarepository/repositories/BEL/). The bone was segmented from the image and solid models developed as part of a previous study.^3^ This geometry formed the basis of models S1a and S1b, where the full pelvic bone was included in S1a and a truncated version was included in S1b. Model S2 was based on a cadaveric right hip, from a 55-year-old male, who died due to the alcoholic cirrhosis of the liver. The pelvic and femur bones were carefully dissected and all soft tissues were removed. The upper and lower parts of the pelvic bone were truncated as the micro-computed tomography (µCT) scanner (µCT 80; Scanco Medical AG, Brüttisellen, Switzerland) was not large enough to accommodate the whole bone. The effect of this truncation of the pelvic bone on the contact mechanics of hip joint was examined using S1a and S1b. The truncated pelvic and femur bones were then imaged sequentially using the µCT scanner at a cubic voxel size of 73.6 µm and energy of 70 kVp, 114 µA. The volumetric µCT data in DICOM format were obtained (Figure 1(a) and (b)) and imported into an image processing software package (ScanIP version 5.1; Simpleware Ltd, Exeter, UK) for segmentation and smoothing. The surfaces of the bones were meshed using three-noded triangular elements which were then exported into a surface-generation software package (Geomagic Studio 11; Geomagic Inc., Research Triangle Park, NC, USA) to produce solid models.

The articular surfaces of the femoral head and acetabular cavity were made perfectly spherical in all segmentation-based models.^3,10,14–16^ For models S1a and S1b, the radii of the acetabular cavity and femoral head were 30.0 and 25.5 mm, respectively. These values were chosen to match the natural radii as closely as possible. The radii of the acetabular cavity and femoral head in model S2, 29.0 and 24.5 mm, respectively, were generated using sphere fitting while having the same radial clearance as models S1a and S1b. A layer of cartilage with uniform thickness of 2 mm was created from the spherical area of the acetabulum and femur for all models.^17^

### Parameterised pelvis and acetabular geometries

**Figure 2.**
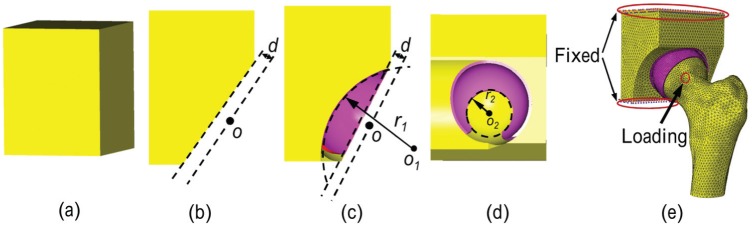
Development of the parameterised model: (a) the cuboid solid model, (b) the generation of the posterior edge of the acetabulum, (c) the generation of the anterior edge of the acetabulum, (d) the generation of the fossa of the cartilage and (e) the FE model and boundary conditions of the parameterised model.

**Figure 3.**
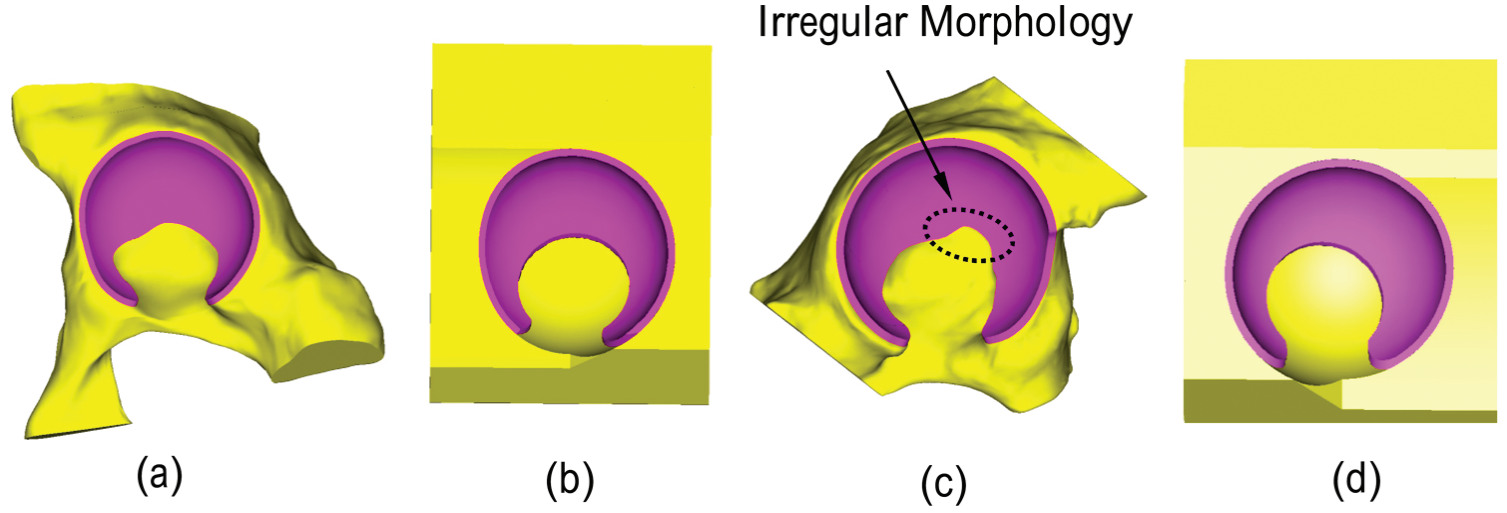
Geometry of acetabulum and cartilage in (a) segmentation-based model S1b, (b) parameterised model P1, (c) segmentation-based model S2 and (d) parameterised model P2. Irregular morphology at the fossa of the cartilage in segmentation-based models.

**Figure 4.**
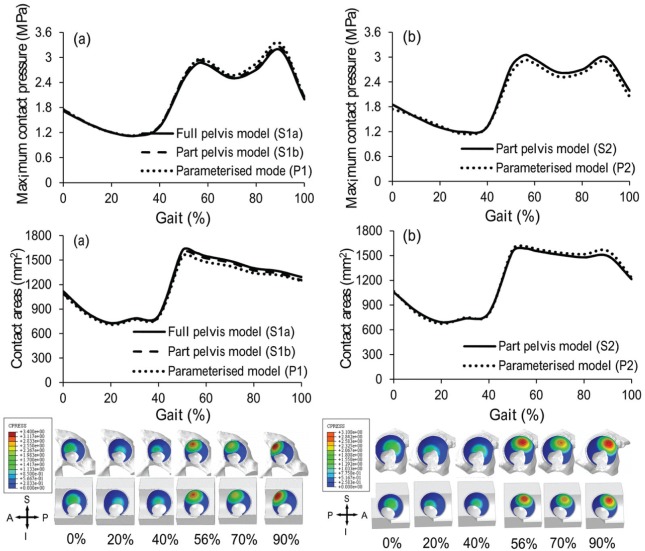
Comparison of maximum contact pressure and contact area and the contact pressure distributions between segmentation-based models and parameterised models for descending stairs for (a) specimen S1 and (b) specimen S2. Models S1 and P1 were from a left hip while models S2 and P2 were from a right hip.

**Figure 5.**
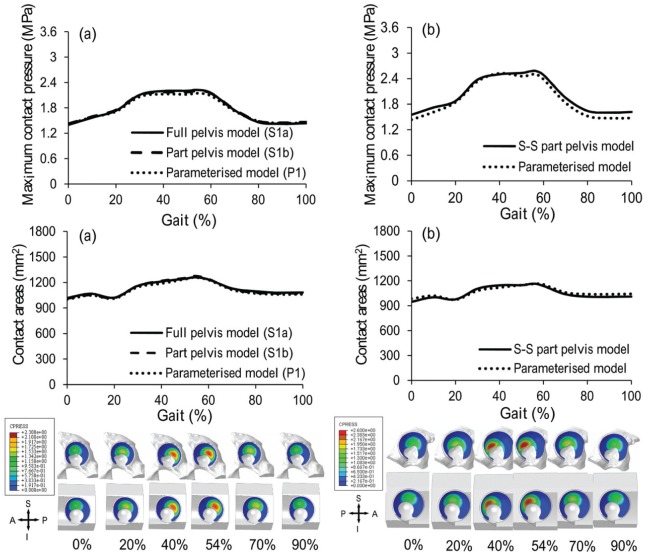
Comparison of maximum contact pressure and contact area and the contact pressure distributions between segmentation-based model and parameterised model for knee bending for (a) specimen S1 and (b) specimen S2. Models S1 and P1 were from a left hip while models S2 and P2 were from a right hip.

Two parameterised models (P1 and P2), each corresponding to one of the segmentation-based models, were generated. The geometries of acetabulum and cartilage in the parameterised models were described using five parameters: the acetabular depth *d*, the centre and radius of the anterior edge cut *o*_1_ and *r*_1_ and the centre and radius of the cartilage fossa *o*_2_ and *r*_2_, as shown in Figure 2. The generation process for the geometries of the acetabulum and cartilage in the parameterised model was as follows: (1) a 70 mm × 85 mm × 80 mm cuboid was built to represent the pelvis (Figure 2(a)); (2) the cuboid pelvis was cut by a spherical surface to create the acetabular cavity. The centre (*o*) and radius of the spherical surface were the same as the ones in the corresponding segmentation-based model; (3) the cuboid pelvis was then cut by a plane with desired inclination angles to generate the posterior edge of the acetabulum. The plane was obtained from the posterior edge of the acetabulum in the segmentation-based model using a best-fit technique.^18,19^ The cup depth *d* was therefore defined as the distances between the plane and the centre of the cavity (Figure 2(b)); (4) the anterior of the cuboid was then cut using a cylinder surface with radius *r*_1_ and centre *o*_1_ in the coronal plane (Figure 2(c)); (5) the cartilage was generated by extruding the articular surface of the cuboid pelvis, which was then cut using a cylinder surface with radius *r*_2_ and centre *o*_2_ in the sagittal plane to create the fossa of the cartilage. The notch of the cartilage was approximated by cutting the inferior edge of the cartilage as the contact areas would not move to this area, and therefore, it has no effect on the cartilage contact mechanics (Figure 2(d)).^3,6^ The values of these parameters in the two parameterised models were measured from the corresponding segmentation-based models using a best-fit technique^18,19^ and are summarised in Table 1. It should be noted that in the parameterised models, the geometry of the acetabulum and cartilage was regular while in the segmentation-based model, some irregular morphology at the edge of acetabulum and cartilage was observed, especially at the fossa edge of the cartilage, as shown in Figure 3.

**Table 1.** Parameter setting calibrated to fit to the two segmentation-based models.

Parameterised models	Segmentation-based equivalent	Acetabulum depth *d* (mm)	Anterior edge		Fossa of cartilage	
			Cut radius *r*_1_ (mm)	Cut centre *o*_1_	Radius *r*_2_ (mm)	Centre *o*_2_
P1	S1b	1.2	43.8	(22.08, −18.78)	16.9	(5.63, −2.35)
P2	S2	0.6	32.6	(15.3, −12.5)	14.2	(6.39, −1.32)

The femur used in the parameterised model was identical to the corresponding segmentation-based model. The acetabular cavity in the parameterised model was considered perfectly spherically. The radius of acetabular cavity, cartilage thickness and the radial clearance between the articular surfaces in the parameterised model were controlled to be the same as the corresponding segmentation-based model.

### FE modelling, boundary and loading conditions, material properties

All the solid models were meshed in I-deas (I-deas, Version 6.1; Siemens PLM Software Inc., Plano, TX, USA) to generate the FE models. The image-based pelvis in the segmentation-based models and cuboid pelvis in the parameterised models as well as the femur in all the models were modelled as two layers, a cancellous bone layer and a cortical shell layer with thickness of 1.5 mm. The segmentation-based FE models comprised approximately 150,000 elements while the parameterised FE models comprised approximately 120,000 elements. Triangular shell elements were used for the cortical bone while tetrahedral and hexahedral elements were used for the cancellous bone and cartilage, respectively. Mesh convergence studies were conducted for each FE model under normal walking activity. Keeping the size of the pelvic bone elements to lower than 3 mm, three models with different levels of mesh density for the pelvic cartilage (with element numbers of approximately 2000, 13,000 and 107,000) and femoral cartilage (with corresponding element numbers of approximately 4000, 15,000 and 124,000) were tested. The results showed convergence trends with respect to the maximum contact pressure and contact area during the whole gait cycle, with the differences in the results between the two finest meshes being within 3% and 1%, respectively. Therefore, the mesh density with approximately 13,000 and 15,000 elements, respectively, on the pelvic and femoral cartilage was selected for all FE models in this study.

In the segmentation-based model S1a, the nodes situated at the sacroiliac joint and about the pubic symphysis were fully constrained. In the models S1b and S2, the nodes located at the truncated area were fully constrained (Figure 1(c)). In the parameterised models P1 and P2, the nodes at the upper and lower planes of the pelvic cuboid were fully constrained (Figure 2(e)). All relative movement was prevented between the pelvic bone and acetabular cartilage, as well as between the femoral bone and femoral cartilage.

In order to apply the load to the FE models without local stress effects, a region of nodes (with radius of approximately 4 mm) was constrained to the load point at the centre of the head, making this region effectively rigid. The centre of the femoral head was then constrained in rotational degrees of freedom. The physiological loadings for seven different human activities, which were measured previously *in vivo* using instrumented total hip prosthesis,^20^ were applied to all the FE models. These activities were as follows: normal walking, ascending stairs, descending stairs, standing up, sitting down, standing and knee bending.^20^ In order to consider the specific direction and orientation of the forces, the resultant hip joint forces were resolved to three components and converted into the FE model coordinate system. During the simulation process, the resultant hip joint forces were discretised into 13 steps and applied to the centre of the femoral head in a quasi-static manner.

All the materials in the FE models were considered as homogeneous, isotropic and elastic, with elastic modulus and Poisson’s ratio values of 17 GPa and 0.3 for cortical bone, 0.8 GPa and 0.2 for cancellous bone^21^ and 12 MPa and 0.45 for cartilage.^22,23^ A frictionless sliding contact formulation was used at the articulating surface between the two layers of cartilage. This assumption was considered reasonable as the friction coefficient between the cartilage surfaces is very low, normally around 0.01–0.02 in the presence of synovial fluid.^3,8,9,24^ The FE analysis was performed using ABAQUS software package (Version 6.9; Dassault Systèmes Simulia Corp., Providence, RI, United States).

### Comparison and data analysis

Each parameterised model prediction was compared to those from the equivalent segmentation-based model with respect to the maximum contact pressures and contact areas for the seven activities. A comparison between the predictions of model S1a and model S1b was also conducted to assess the influence of the amount of pelvis included in the model on the cartilage contact mechanics. The contact areas were calculated by summing individual element areas for only those elements that had nodal pressures greater than 0.1 MPa.^8,25^ In order to highlight the effect of the geometry on the cartilage contact mechanics, some parameters were controlled for both the segmentation-based models and parameterised models, as shown in Table 2.

**Table 2.** Controlled and variable parameters in the segmentation-based and parameterised model.

Parameters	Models	
	Segmentation-based	Parameterised
Pelvic bone surface (away from acetabular)	Consequence of scan-based geometry	Approximated to a cuboid
Acetabular cavity surface	Controlled (spherical)	Controlled (spherical)
Femoral head surface	Controlled (spherical)	Controlled (spherical)
Cartilage thickness	Controlled (2 mm)	Controlled (2 mm)
Inclination angle	Controlled (63°)	Controlled (63°)
Anteversion angle	Controlled (15°)	Controlled (15°)
Radial clearance	Controlled (0.5 mm)	Controlled (0.5 mm)
Femoral head radius	Based on best-fit cut	Based on best-fit cut
Acetabular cavity radius	Based on best-fit cut	Same with segmentation-based model
Acetabular depth	Consequence of scan-based geometry	Set based on measurements using plane best-fit technique
Fossa centre and radius	Consequence of scan-based geometry	Set based on measurements using spherical best-fit technique
Radius and centre of anterior edge cut	Consequence of scan-based geometry	Set based on measurements

## Results

The data associated with this paper (additional method details, segmentation geometries, model input files and results) are openly available from the University of Leeds Data Repository (http://doi.org/10.5518/3).

### Pelvic bone sensitivity

**Figure 6.**
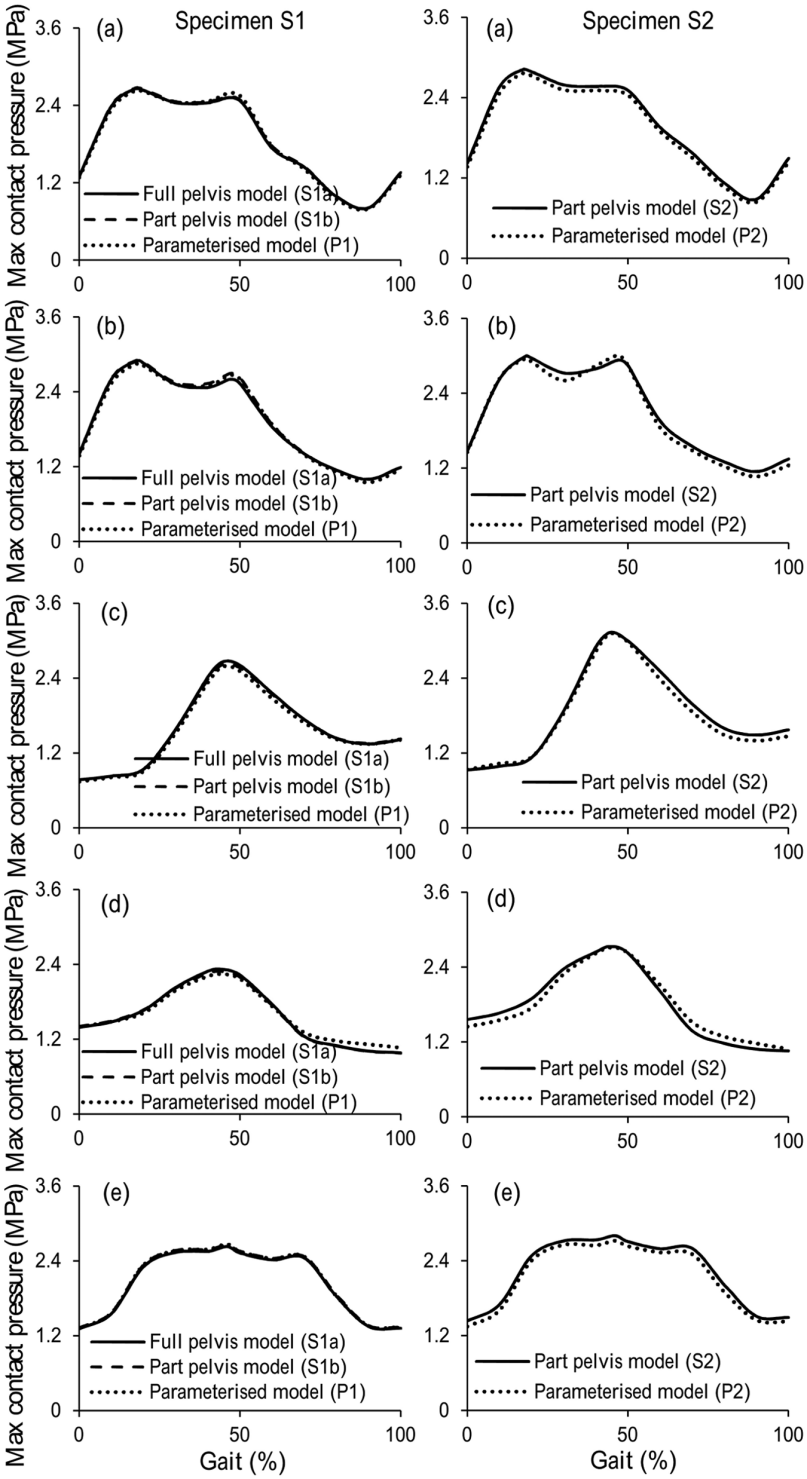
Comparison of maximum contact pressures between segmentation-based model and parameterised model for (a) normal walking, (b) ascending stairs, (c) standing up, (d) sitting down and (e) standing for the two pelvic bone specimens.

**Figure 7.**
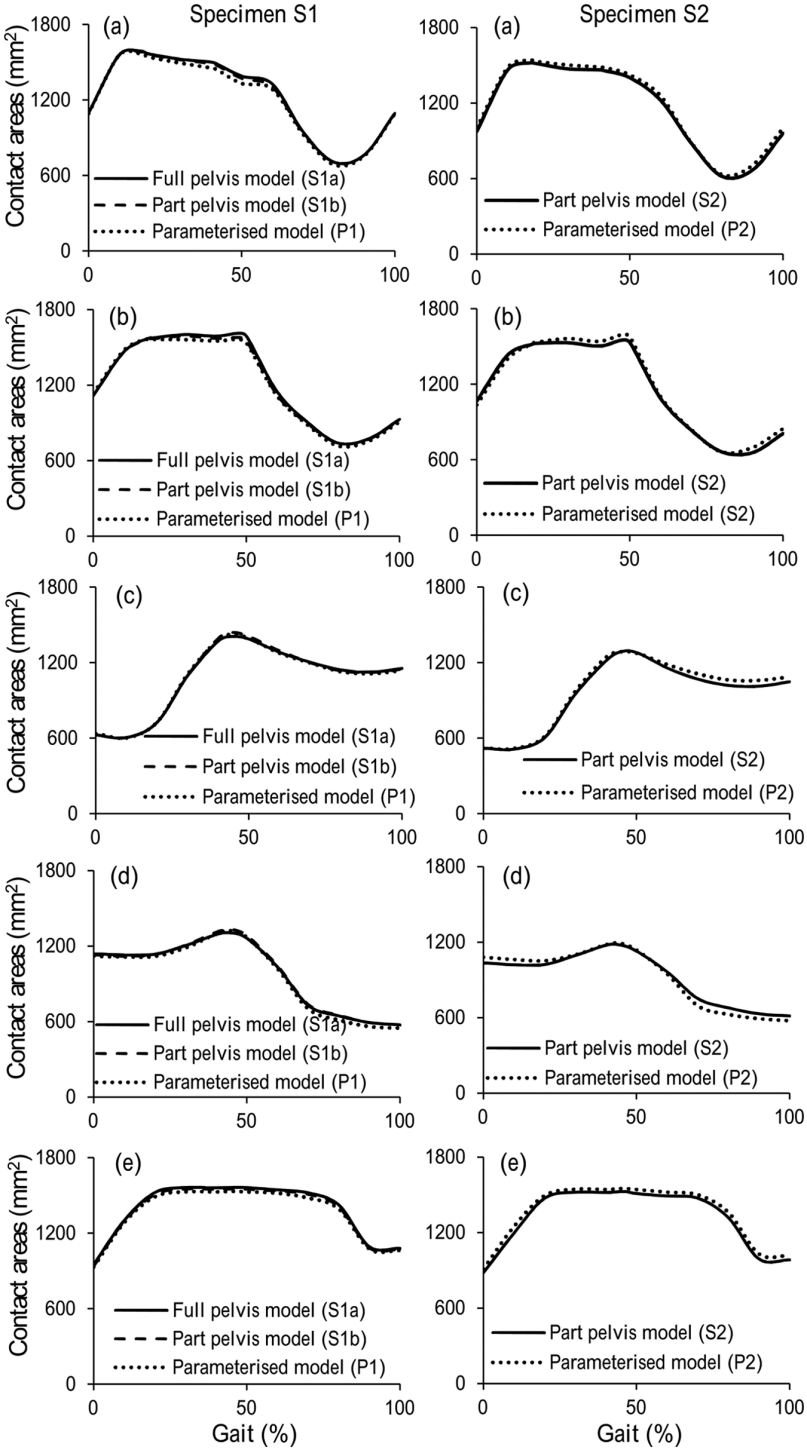
Comparison of contact areas between segmentation-based model and parameterised model for (a) normal walking, (b) ascending stairs, (c) standing up, (d) sitting down and (e) standing for the two pelvic bone specimens.

When comparing the segmentation-based models with full pelvis (S1a) and with part pelvis (S1b), the maximum differences were 5.3% and 2.5% for contact pressure and contact area, respectively. These occurred at 47% gait cycle of ascending stairs activity (Figures 4(a), 5(a), 6 and 7).

### Comparison of parameterised and segmentation-based models

The results predicted by the parameterised model and the segmentation-based model matched well for both hip specimens and across all the activities, with maximum differences of 10.1% and 8.3% for cartilage contact pressure and contact area, respectively. For specimen 1 (comparison of S1b and P1), the maximum difference was for the ‘descending stairs’ at 90% of the gait cycle, where the contact area is approaching the superior–anterior edge of the cartilage (Figure 4). For specimen 2 (comparison of S2 and P2), the maximum difference was for the ‘knee bending’ activity at 90% of the gait cycle, where the contact area is near the superior–posterior edge of the fossa of the cartilage (Figure 5). The comparative results from the remaining five activities can be seen in Figures 6 and 7.

The same trends in contact pressure and contact areas were predicted between the segmentation-based models (S1b and S2) for each activity, with the exception of the descending stair case. Here, model S1b showed a consistent downwards trend in contact area, whereas S2 included a peak at 90% of the gait cycle. However, the trends predicted by the segmentation-based models for the two pelvis specimens were reflected in the matching parameterised models in each case, including the descending stair case (Figure 4).

## Discussion

This study developed an initial, novel methodology for generating parameterised geometric models of the human acetabulum based on measurements from three-dimensional images and provided evidence of its ability to replicate subject-specific results from established segmentation-based approaches. The value of this approach is that it enables the effect of individual geometrical features to be tested in isolation, through the rapid generation of models with controlled geometric variations. This is of special significance as the geometries of the acetabulum and the cartilage are important factors contributing to the abnormal mechanical conditions in the joint (such as impingement)^26–28^ and abnormal function of the hip (such as dysplasia).^29–32^ Although these features can be analysed qualitatively using subject-specific segmentation-based models,^7,9^ quantitative analysis is difficult to achieve in these complicated and time-consuming models. Parameterised models are an important step to allow these features to be described and analysed individually and tested quantitatively. For example, in the parameterised models in this study, by changing the acetabulum depth and cartilage coverage (i.e. the cut radius and cut centre of the anterior edge and cartilage fossa), the biotribology of the hip across the normal population and different group of patients could be quantified and analysed. More importantly, these parameterised models have the potential, as an important protocol, to be generated rapidly and automatically based on measurements from medical images, which is the goal of our future studies.

The premise of the study is that the parameterised models developed from the micro-CT images could be used to investigate the cartilage contact mechanics in the hip. Hence, direct comparisons were conducted between parameterised models and segmentation-based models which were developed directly from the three-dimensional micro-CT images of two human hip specimens. The preliminary results showed that the parameterised modelling system can replicate trends in cartilage contact mechanics found in the individual segmentation-based model, for all seven activities considered. The major differences between the parameterised and segmentation-based models lay in the geometry of the pelvic bone and the shape of the acetabular cartilage boundary. Therefore, differences seen in the cartilage contact mechanics can clearly be attributed to one of these features. Where the peak differences were seen, they can be linked to a difference in contact area due to simplification of the cartilage edge shape (Figures 4 and 5).

The segmentation-based models of the two individuals predicted consistent trends in the cartilage contact pressures and contact areas for most activities, with the exception of the descending stair case, where different trends were predicted in the second half of the cycle (Figure 4). At this point, the contact area is close to the outer anterior edge of the cartilage. It is encouraging that the parameterised model was able to replicate these contact mechanics trends, seen in each individual segmentation-based case, despite the relative simplicity of the anterior outer edge cut. This ability is significant as this portion of the descending stair cycle includes the highest contact pressures over all activities tested. Since the parameterised models are fully controlled, differences between model geometries, causing differences in results, can be easily identified. Through a comparison of the two parameterised models for descending stairs, the difference in anterior edge position can be identified as the source of the difference in contact area trends. This information would be less straightforward to extract given only the segmentation-based models.

In this study, the maximum cartilage contact pressures were predicted to be 2.4–3.5 MPa for different activities when a spherical articulating surface and uniform cartilage thickness as well as a human body weight of 80 kg were considered. These contact pressures were found to be in good agreement with a previous FE study,^33^ in which the cartilage contact pressures were reported to be 2.5–3.5 MPa for different activities when the same cartilage thickness and radial clearances were considered and the same kinematic and loading conditions were applied, providing some verification for the segmentation-based model and parameterised model in this study. Yoshida et al.^16^ developed dynamic discrete element models to predict hip joint contact pressures and reported peak values of 3.26, 3.77 and 5.71 MPa during walking, descending stairs and ascending stairs, respectively, which are also comparable with the results predicted in this study. Clearly, the assumptions of spherical geometry and constant thickness of cartilage underestimated the cartilage contact stress in the real human joint, as reported in previous FE studies^7–9^ and experimental studies.^34–36^ However, as the main purposes of this study were to develop and evaluate a parameterised model of the hip by comparison with a corresponding segmentation-based model, rather than directly validating an FE model against experiment, such assumptions were considered to be justified.

This study was a first step towards a parameterised model of the human hip and as such the models used were highly simplified and controlled. The cartilage was assumed to be a homogeneous, isotropic and linear elastic material with a uniform thickness, rather than a material exhibiting biphasic behaviour.^1,3^ Analysis of the contact mechanics is therefore limited to instantaneous behaviour, and although comparisons of contact mechanics between different geometric cases have merit for ranking the effect of geometric features, magnitudes are not representative of the *in vivo* case. Future studies using the parameterised hip model could therefore incorporate more sophisticated materials for the cartilage, such as biphasic properties,^1,3,10,33^ and more parameters to represent the shape and thickness of the cartilage,^5,7,25^ in order to achieve results more in line with the *in vitro* and *in vivo* performance.

In all the models, the cortical bone was represented by shell elements with a uniform theoretical thickness and the subchondral bone was not differentiated from the cancellous bone. A uniform elastic modulus was used for both the cortical and cancellous bone. In addition, different numbers of element were used for the pelvic bone in the segmentation-based model and parameterised model due to the different geometries, although same element numbers and types were adopted for the cartilage components. However, due to the extremely high modulus of the bony components relative to the cartilage, sensitivity to changes in the bone material and properties and the effect of different element numbers for the pelvic bone in the two types of models are expected to remain low. This was supported by comparing the models S1a and S1b in this study, which demonstrated that reducing the amount of pelvic bone represented in the segmentation-based model had little effect on the contact pressure and area. These differences were of an equivalent magnitude or smaller than the effect of changing to a parameterised geometry (comparison of S1b and P1).

The labrum was not included in any of the models, and therefore, its effects are not considered in this comparison of modelling approaches.^37,38^ As the position of the outer edge of the cartilage has been identified as influencing contact mechanics trends, the addition of the labrum is arguably the next step in parameterised model development.

In order to draw conclusions about the wider applicability of this parameterised system, it is necessary to perform comparisons with a larger set of segmentation-based models. Although a minimal set of parameters is desirable, more could be added where necessary using the current approach to capture features present in the wider population. Although the parameterised models in this study were developed from three-dimensional CT images for the purpose of direct comparison with the corresponding segmentation-based models, it is envisaged that such a modelling system could be applied to a wider cohort of patients from two-dimensional radiographs in future studies, provided that methods of effectively capturing the geometric parameters of acetabulum and cartilage were developed.^39,40^ The segmentation-based models in this study were simplified in key ways, such as the creation of uniform cartilage thickness, so that specific comparisons could be made with the parameterised models. As the parameterised modelling system develops, it should be tested against progressively less controlled segmentation-based models with increasingly realistic features. The results of this preliminary study are encouraging and give confidence in a parameterised approach, which has the potential to allow testing of isolated geometric features whose variation can be captured from simple clinical measurements.
